# Optimal Choice of Radiotherapy in Locally Advanced, Low Rectal Cancer: A Propensity Matched Analysis Comparing Short‐Course Radiation With Long‐Course Chemoradiation From a Tertiary Cancer Center

**DOI:** 10.1002/jso.70019

**Published:** 2025-06-26

**Authors:** Tejas Vispute, Devesh S. Ballal, Raj Kapadia, Ashwin Desouza, Ankit Sharma, Mufaddal Kazi, Akshay Baheti, Vikas Ostwal, Avanish P. Saklani

**Affiliations:** ^1^ Division of Colo‐Rectal and Peritoneal Surface Oncology, Department of Surgical Oncology Tata Memorial Hospital, Homi Bhabha National Institute Mumbai India; ^2^ Department of Surgical Oncology Manipal Hospital Bangalore India; ^3^ Department of Radiology Tata Memorial Hospital, Homi Bhabha National Institute Mumbai India; ^4^ Department of Medical Oncology Tata Memorial Hospital, Homi Bhabha National Institute Mumbai India

**Keywords:** long course chemoradiation, outcomes, rectal cancer, short course radiation

## Abstract

**Aim:**

To compare oncological outcomes of short‐course radiation therapy (SCRT) versus long‐course chemoradiation (LCRT) in patients with low rectal cancer, particularly in a high‐volume center with expertise in extended total mesorectal excision (TME).

**Methods:**

This was a single‐institution, retrospective propensity‐matched study using a prospectively maintained database. Patients with low rectal cancer (≤ 5 cm from the anal verge) who underwent neoadjuvant radiation (SCRT or LCRT) followed by TME between January 2014 and January 2021 were included. A 3:1 propensity score match was performed based on key clinical variables. Patients with metastatic disease or prior pelvic radiation were excluded. SCRT (25 Gy in 5 fractions) ± chemotherapy was followed by immediate or delayed surgery, while LCRT (50–50.4 Gy in 25–28 fractions) was given with capecitabine ± chemotherapy, followed by surgery. Extended resections were performed as indicated.

**Results:**

After matching, 466 LCRT and 157 SCRT patients were analyzed. Three‐year disease‐free survival (DFS) was similar (62% LCRT vs. 64% SCRT, *p* = 0.8), with no significant differences in overall survival (OS), local recurrence‐free survival (LRFS), pathological complete response (pCR: 18% vs. 20%, *p* = 0.5), or circumferential resection margin (CRM) positivity (6.4% vs. 10%, *p* = 0.12). Complication rates and local recurrence were also comparable. However, among clinical T4 tumors, SCRT was associated with significantly lower 2‐year DFS (41.2% vs. 58.7%, *p* = 0.03) and a trend toward worse OS.

**Conclusion:**

SCRT provides comparable oncological outcomes to LCRT in low rectal cancer when appropriately selected. However, in clinical T4 tumors, LCRT appears.

**Abbreviations**
CD: Clavien‐Dindo complication
CRM: circumferential resection margin
LCRT: long‐course chemoradiation
pCR: pathological complete response
RT: radiotherapy
SCRT: short‐course radiation
TME: total mesorectal excision
TNT: total neoadjuvant therapy

## What Does This Paper Add to the Literature?

This study demonstrates that short‐course radiation offers comparable oncological outcomes to long‐course chemoradiation in low rectal cancer, except in clinical T4 tumors where long‐course treatment is superior—highlighting the value of SCRT in selected patients, particularly in resource‐limited settings.

## 1 Introduction

Preoperative radiation followed by total mesorectal excision (TME) has long been considered the standard of care for locally advanced rectal cancer. While the popularization of total neoadjuvant therapy (TNT) has led to preoperative chemotherapy being delivered to many patients, the choice of optimal radiation modality (long‐course chemoradiation (LCRT) or short‐course radiation (SCRT)) remains a contentious issue.

In the pre‐TNT era, the choice between LCRT as per the German rectal cancer trial [1] and SCRT with consolidation chemo as described in the trial by Bujko et al. [2, 3] was difficult to make. With the publication of the initial results of the RAPIDO trial [4] (that showed a 7.5% improvement in distant metastasis‐free survival) which led to the widespread popularization of TNT and SCRT with consolidation became universally accepted as standard of care. However, the publication of the long‐term results of the RAPIDO trial [5] which showed increased local recurrence and reduction in the quality of TME specimens in the TNT arm, SCRT and consolidation chemotherapy has fallen out of favor because of concerns of more difficult surgery due to radiation induced fibrosis and loss of tissue planes. This coincided with the widespread adoption of LCRT and consolidation chemotherapy after the publication of the OPRA trial [6] which demonstrated successful organ conservation in almost 50% of moderate‐risk rectal cancers and hence the question of whether SCRT still has a role in low rectal cancers in the present day.

It's worth noting that the numerous advantages of SCRT, including shorter treatment duration, improved patient compliance, reduced costs, and shorter intervals between RT and systemic therapy deliveries, which contributed to its widespread popularity, continue to be highly relevant, particularly in resource‐constrained countries like India, where access to tertiary care is limited, and existing radiotherapy facilities are operating at full capacity. It is also worth noting that the incidence of adverse histologies (mucinous and signet ring cancers) is much higher and stage of presentation is later in these resource‐constrained settings [7]. This study was conceived to study whether SCRT is a viable treatment option in the current era, in a high‐volume center where extended TME is performed routinely with a low incidence of positive CRM even in low rectal cancers with involved MRF.

## 2 Materials and Methods

### 2.1 Study Design

Single institute retrospective study using data from a prospectively maintained database of patients undergoing mesorectal excision (abdominoperineal resection, intersphincteric resection or pelvic for low rectal cancer (defined as cancer within 5 cm of the anal verge on digital rectal exam and MRI scan) after receiving neoadjuvant radiation between January 2014 to January 2021. Patients with metastatic disease, those who had received prior pelvic radiation therapy were excluded from analysis. Further, patients in whom a watch‐and‐wait approach was employed were excluded from the study as the total number of these patients was small.

**Figure 1:**
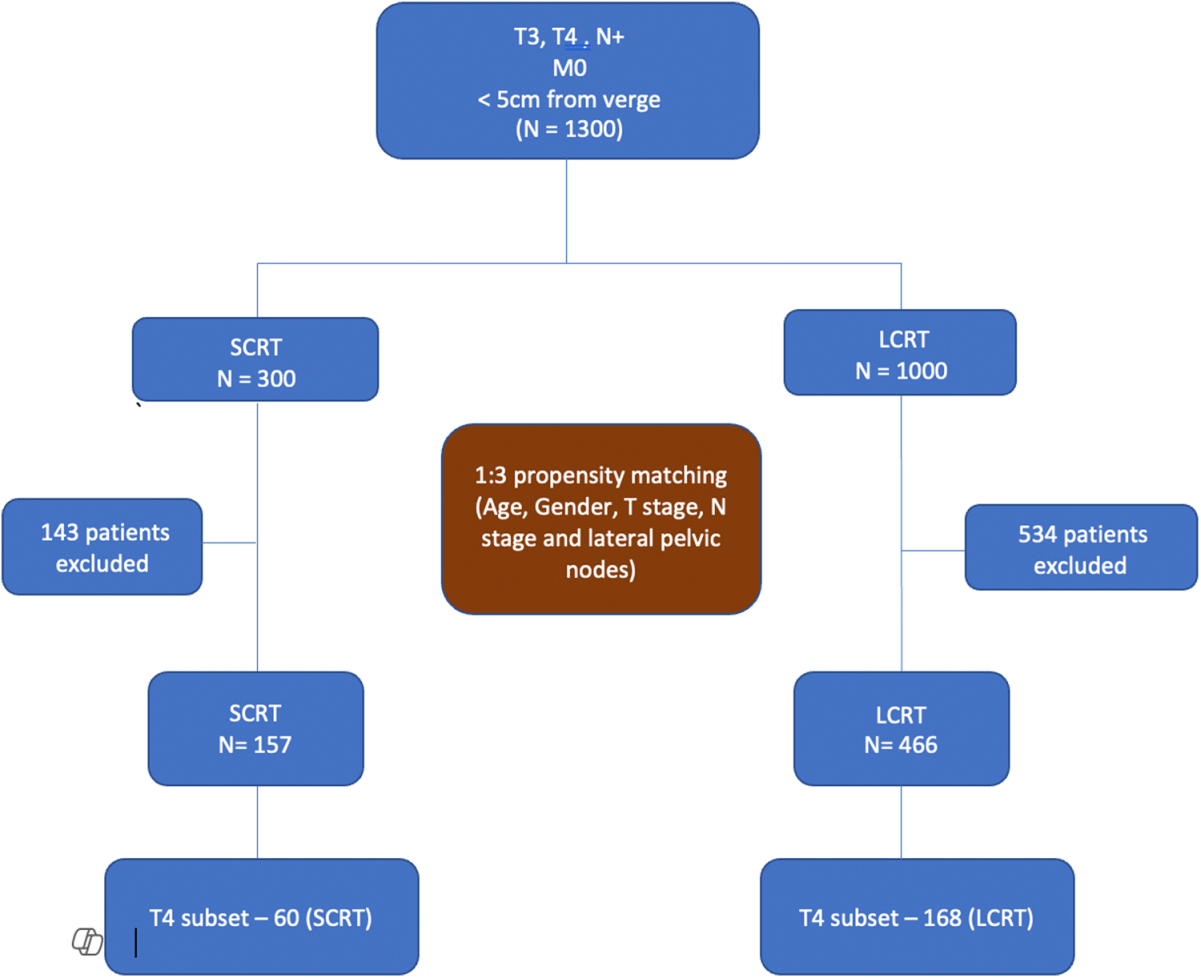
Consort diagram depicting selection of patients.

The flow of patients though the study is depicted in the consort diagram in Figure 1. Patients in SCRT group received radiation in the dose of 25 Gy in five fractions with or without chemotherapy followed by immediate or delayed surgery. Patients in LCRT group received 50–50.4 Gy in 25–28 fractions with Capecitabine with or without chemotherapy, followed by surgery. All treatment decisions were taken by a multidisciplinary tumor board and were based on available guidelines at the time. Patients with persistent organ involvement (ycT4) were treated with either beyond‐TME or exenteration surgeries.

MRI was performed using a 1.5 T MRI without an endorectal coil and without contrast. Diffusion‐weighted sequences were performed for all post‐neoadjuvant MRI scans. MRI scans were reported by one of three dedicated GI radiologists. MRF < 1 mm was considered as involved on MRI.

The decision on type surgery performed was left to treating surgeon. Patients who had positive lateral pelvic nodes after neoadjuvant therapy as defined by the Ogura criteria [8], underwent a lateral pelvic lymph node dissection on the involved side.

The decision on whether to use a total neoadjuvant approach was decided by the multidisciplinary tumor board.

A standardized follow‐up policy requiring 3 monthly follow‐ups for the first 2 years and 6 monthly follow‐ups for the next 3 years with CEA levels, CT scans, and colonoscopy being ordered as per the NCCN guidelines available at the time.

### 2.2 Propensity Score Matching (PSM)

To reduce the bias due to the retrospective nature of the study, propensity score matching was done. Logistic regression was used to calculate propensity scores for each patient based on age, gender, CEA level, T, N status, and presence of lateral nodes. PSM was then done at a 3:1 ratio and caliper of 0.25.

### 2.3 End Points

The primary end point was the disease‐free survival (DFS) with secondary end points being overall survival, local‐recurrence‐free survival (LRFS), rate of pathological complete response and CRM positivity. DFS was defined as time from surgery to disease recurrence or death due to any cause or last known follow up whichever was earlier. Overall Survival (OS) was defined as time from surgery to death due to any cause or last known follow up whichever was earlier. LRFS was defined as the time from surgery to local disease recurrence or death due to any cause or last known follow up whichever was earlier.

### 2.4 Statistical Analysis

SPSS v30.0 by IBM was used for statistical analysis.

Categorical values are presented as percentages and frequencies and compared using the Chi‐squared test. Continuous variables are presented are medians with interquartile range and compared using the Mann‐Whitney U test. Survival curves were plotted using the Kaplan‐Meier method and compared using the log‐rank test. Follow‐up duration was calculated using the reverse Kaplan‐Meier method and presented as median duration with 95% confidence intervals.

### 2.5 Ethics

Informed consent was obtained from each patient during treatment and surgery. The study protocol strictly complied with the ethical standards set by the institutional research committee and the 1964 Helsinki Declaration, as well as its subsequent amendments. Notably, due to the retrospective nature of the study and the use of anonymized data, formal approval from the institutional review board was not required.

## 3 Results

The initial database contained 1300 low rectal cancer patients (1000 receiving LCRT and 300 receiving SCRT) which were then propensity matched as depicted in the consort diagram in Figure 1. After propensity matching, there were 466 and 157 patients in the LCRT and SCRT groups, respectively. The demographic and baseline clinical variables were evenly matched after propensity matching, as presented in Table 1, except for BMI, where a slightly higher median BMI was noted in the LCRT group. The incidence of nodal positivity was 90% and similar in both groups, with an incidence of lateral node positivity of 25%. A majority of patients had MRF positivity on the preoperative scan, with no difference between both groups (57% and 62% in the LCRT and SCRT groups, respectively, *p* = 0.094). There was a significant difference in type of surgical approach (higher percentage of minimally invasive surgeries in the SCRT group) and type of surgery (more sphincter‐saving surgeries in the SCRT group) as depicted in Table 1.

**Table 1:** Baseline and operative details after propensity score matching.

	After propensity matching		
	LCRT	SCRT	*p* value
	*N* = 466	*N* = 157	
Age in years (IQR)	45 (34–54)	44 (34–57)	0.769
BMI in kg/m^2^ (IQR)	23 (21–25)	21 (19–24)	0.033
Gender			0.9
Male	316 (68%)	107 (68%)	
Female	149 (32%)	36 (32%)	
Median CEA (IQR)	4.6 (2.6–15.0)	4.0 (2.1–12.6)	0.8
Clinical T stage			0.8
T2	35 (7.5%)	8 (5%)	
T3	253 (54.5%)	80 (51%)	
T4	178 (38%)	69 (44%)	
Clinical N stage			0.841
N0	53 (11%)	16 (10%)	
N1	218 (47%)	75 (48%)	
N2	195 (42%)	66 (42%)	
Clinical Lateral Node positivity	116 (25%)	42 (25%)	0.8
Baseline MRF involvement	265 (57%)	98 (62%)	0.094
Signet Histology	79 (17%)	34 (22%)	0.19
Duration between RT and Surgery in weeks (IQR)	10 (8–20)	14 (8–23)	0.017
Surgery done			0.006
APR	288 (62%)	86 (55%)	
ISR	92 (20%)	39 (25%)	
Total pelvic exenteration	68 (14%)	26 (16%)	
Posterior Exenteration	18 (4%)	6 (4%)	
Surgical approach			< 0.001
Open	196 (42%)	34 (22%)	
Laparoscopic	212 (46%)	102 (65%)	
Robotic	58 (12%)	20 (13%)	
LPLND done	71 (15%)	24 (15%)	0.9
Neoadjuvant chemotherapy	43 (9%)	125 (80%)	< 0.001
Adjuvant chemotherapy	303 (65%)	77 (44.6%)	< 0.001

Abbreviations: APR, abdominoperineal resection; CEA, carcinoembryonic antigen; LCRT, long course chemoradiotherapy; LPLND, lateral pelvic lymph node dissection; MRF, mesorectal fascia; ISR, intersphincteric resection; SCRT, short course radiotherapy.

There was a significant difference in the utilization of preoperative chemotherapy (9% as opposed to 80% in the LCRT and SCRT groups, respectively, *p* < 0.001) and the median duration from radiation to surgery (10 weeks and 14 weeks in the LCRT and SCRT groups, respectively, *p* = 0.017).

The incidence of major complications (> Clavien‐Dindo grade 2) was low and similar between both groups (5.8% and 8.3% in the LCRT and SCRT groups, respectively, *p* = 0.27) and there was no difference between the rates of pCR (18% and 20% in the LCRT and SCRT groups, respectively, *p* = 0.5) or CRM positivity (6.4% and 10% in the LCRT and SCRT groups, respectively, *p* = 0.12) in both groups as depicted in Table 2 which displays the pathological and oncological outcomes. There was however a significant difference in the median follow‐up duration between the groups (51 months and 21 months in the LCRT and SCRT groups, respectively, *p* < 0.01)

**Table 2:** Pathological and long‐term outcomes.

	After propensity matching		
	LCRT	SCRT	*p* value
	*N* = 466	*N* = 157	
Complication ≥ CD3a	27 (5.8%)	13 (8.3%)	0.27
CRM positive	30 (6.4%)	16 (10%)	0.12
Pathological node positivity	141 (30%)	52 (33%)	0.32
Pathological complete response	84 (18%)	32 (20%)	0.5
Recurrence	169 (36%)	44 (28%)	0.06
Local	37 (8%)	8 (5%)	
Distant	136 (29%)	34 (22%)	
Median follow up (95%CI)	51 (45–56)	21 (14–27)	< 0.01
	44 (49–48)		
3‐year OS	76.6%	72.1%	0.7
3‐year DFS	62%	64%	0.8
3‐year LRFS	93%	91.1%	0.9

Abbreviations: DFS, disease‐free survival; LCRT, long‐course chemoradiotherapy; LRFS, local recurrence‐free survival; OS, overall survival; SCRT, short‐course radiotherapy.

**Figure 2:**
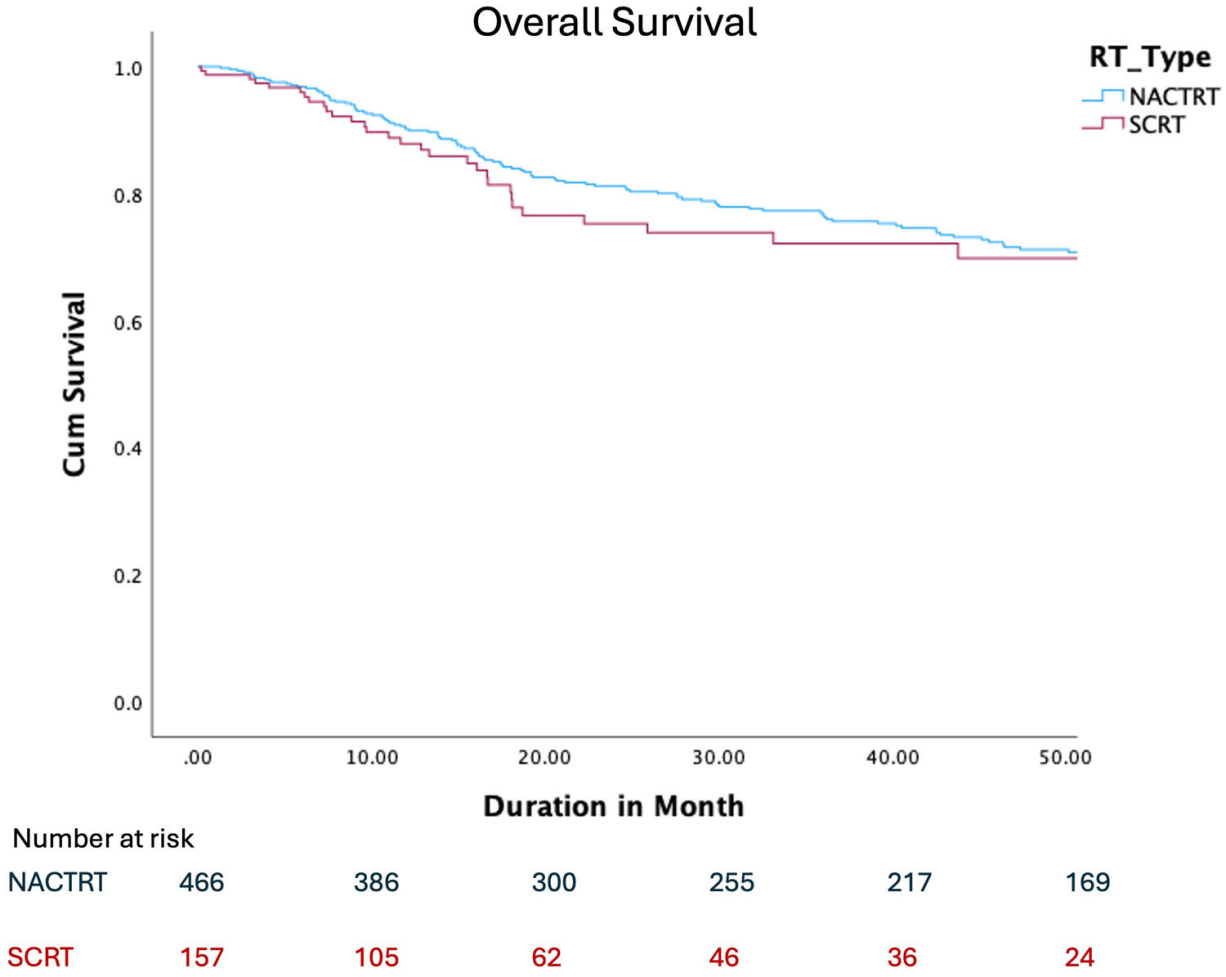
Overall survival of matched cohort.

**Figure 3:**
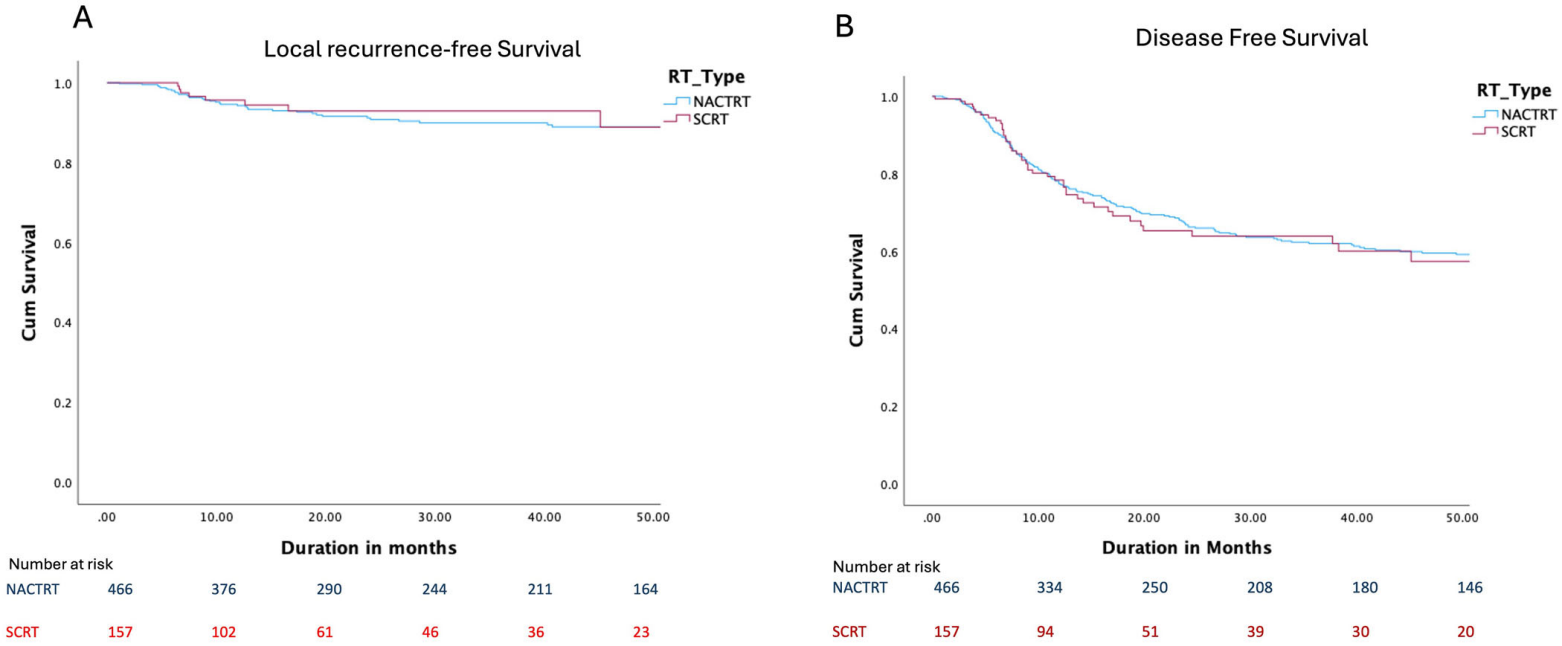
Locoregional recurrence (panel A) and disease‐free survival (panel B) of matched cohort.

The long‐term oncological outcomes were also comparable with a similar OS as depicted in Figure 2, DFS and LRFS as depicted in Figure 3. The rates of local recurrence were also similar between both groups (8% and 5% in the LCRT and SCRT groups, respectively, *p* = 0.12).

**Figure 4:**
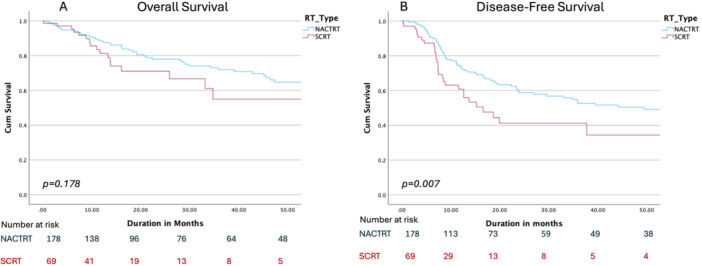
Overall survival (panel A) and disease‐free survival (panel B) of T4 subset only.

Subgroup analysis of only clinical T4 cancers revealed similar CRM positivity, pathological nodal positivity, and pathological complete response rates between LCRT and SCRT groups as shown in Table 3. However, in cT4 patients SCRT was associated with a statistically significant detriment in DFS (2‐year DFS 58.7% and 41.2% in the LCRT and SCRT groups, respectively, *p* = 0.007) and a numerically worse overall survival, albeit not statistically significant (2‐year OS 77% and 71% in the LCRT and SCRT groups, respectively, *p* = 0.178). The OS and DFS of the T4 subset are depicted in Figure 4.

**Table 3:** Pathological and long‐term outcomes of cT4 subset.

	After propensity matching		
	LCRT	SCRT	*p* value
	*N* = 178	*N* = 69	
CRM positive	14 (7.9%)	9 (13%)	0.209
Pathological node positivity	68 (38.2%)	28 (40.1%)	0.32
Pathological complete response	22 (12.4%)	10 (14.5%)	0.647
Recurrence	71 (39.9%)	31 (44.9%)	0.470
Local	11 (8%)	4 (5%)	
Distant	40 (29%)	18 (22%)	
Median follow‐up (95%CI)	41 (16–73)	16 (8–33)	< 0.01
2‐year OS	77%	71%	0.178
2‐year DFS	58.7%	41.2%	0.03

Abbreviations: DFS, disease‐free survival; OS, overall survival; LCRT, long‐course chemoradiotherapy; LRFS, local recurrence free survival; SCRT, short‐course radiotherapy.

## 4 Discussion

The present study does not show any difference between the two arms in terms of 3‐year overall survival, DFS or local recurrence‐free survival. While there was a trend towards fewer recurrences in the SCRT arm, this could be attributed to a shorter median follow‐up duration in the SCRT arm (51 months and 21 months in the LCRT and SCRT groups, respectively, *p* < 0.01). There was also no statistically significant difference between the rates of pathological CRM positivity between both arms. These findings align with the results of Bujko et al [3], which demonstrated a similar OS, DFS and incidence of local or distant failure at 7 years of follow‐up (in a cohort of with 57% of patients with low‐rectal cancer). However, the long‐term results of the RAPIDO trial [5] which showed a fivefold increase in the rates of breached MRF and higher local recurrence rate in the SCRT arm contradict our findings. A possible explanation for this is that longer duration between RT and surgery in the RAPIDO trial (with only 24% of patients having rectal cancers) wherein 17 weeks of consolidation chemotherapy was used (and time to surgery was 25 weeks from randomization), as opposed to the 5 weeks used in the trial by Bujko et al. Most patients (80%) in our study in the SCRT arm received 4 cycles of consolidation chemotherapy with a median time to surgery of 14 weeks. One of the hypotheses for the increased rates of breached mesorectum and poor local control rates in the RAPIDO rial was the long interval between SCRT and surgery in the experimental arm in the RAPIDO trial. Delays of surgery greater than 11 weeks have been shown to increase operative difficulty and morbidity [9]. Similarly, the Stellar trial [10] which included high‐risk cancers (49% of patients had low rectal cancers) and only 4 cycles of consolidation chemotherapy showed a higher rate of pCR in the SCRT‐consolidation chemotherapy group with no difference in locoregional recurrence or DFS.

Our subset analysis of clinical T4 tumors indicates that the role of SCRT in T4 cancers warrants further investigation, as it was associated with inferior DFS (2‐year DFS 41.2% vs. 58.7%, *p* = 0.03) and a trend towards worse overall survival, although the latter was not statistically significant (2‐year OS 71% vs. 77%, *p* = 0.178). Importantly, the rates of CRM positivity, nodal disease, and pCR remained comparable in the T4 subgroup. These findings raise the possibility that while SCRT may provide equivalent outcomes in lower‐risk or T3 tumors, patients with T4 disease may derive greater benefit from LCRT. It's also noteworthy that the recent update of the RAPIDO trial [5] (which included 31% of patients with cT4 disease) revealed that SCRT and consolidation chemotherapy were associated with a 2.34 times higher risk of locoregional recurrence compared to standard LCRT. It would be intriguing to analyze the cT4 subgroup of the RAPIDO trial to determine the extent to which this subgroup contributed to the increased locoregional failure in the experimental arm.

In our study, there was no difference in pCR between the two arms. Two possible explanations for this could be the lower number of consolidation chemotherapy cycles employed in our study and the high proportion of patients with signet cell cancers (20% of patients in our study has signet ring cell cancers as opposed to 10% poorly differentiated with no signet ring cancers in the RAPIDO study). The use of only four cycles of consolidation chemotherapy in our study is an outlier as opposed to most other TNT trials which in which more chemotherapy was given (pCR rates of 28% in the RAPIDO trial after 6 cycles of consolidation chemotherapy). It is also worth noting that in our study all patients with persistent lateral pelvic nodes underwent a lateral pelvic lymph node dissection, unlike in the RAPIDO study where 15% of patients had suspicious lateral pelvic lymph nodes, but no lateral pelvic lymph node dissection was done.

It's worth noting that both groups in our study included a substantial portion of extremely high‐risk, “ugly” rectal cancers with baseline suspicious lateral lymph nodes present in 25% of patients, baseline MRF involvement in more than 55% of patients and with signet cell histology present in 17% and 22% in the LCRT and SCRT groups respectively. Despite this very high‐risk cohort being treated in both arms, the pathological CRM positivity was acceptable (6.4% and 10% in the LCRT and SCRT arms respectively, *p* = 0.32) with a similar pathological complete response rate of 18% and 20% in the LCRT and SCRT arms respectively (*p* = 0.5). Despite a high proportion of extended TME and beyond TME surgeries (33.7% and 38.2% in the LCRT and SCRT arms respectively), there was a similar and acceptable rate of major postoperative complications (incidence of complications ≥ Clavien‐Dindo grade 3a of 5.8% and 8.3% in the LCRT and SCRT arms respectively, *p* = 0.27). These findings resonate with the findings of other trials that revealed comparable postoperative morbidity and short‐term outcomes between SCRT and LCRT [11, 12, 13].

Despite propensity matching of known preoperative confounders, there was a statistically significant difference in the rates of sphincter preservation surgery (20% and 25% in the LCRT and SCRT arms respectively, *p* = 0.006) and utilization of minimally invasive surgery (58% and 78% in the LCRT and SCRT arms respectively, *p* < 0.001). An explanation for this could be that patients with locally advanced and bulky tumors that required more extensive downstaging to facilitate surgery were more likely to be treated with LCRT.

The adoption of SCRT in resource‐limited settings is further supported by its logistical advantages—shorter treatment duration, lower cost, and improved compliance. While these benefits are particularly relevant in countries like India, our data suggest that SCRT should be cautiously applied in patients with clinical T4 tumors until prospective validation is available. With the available data and resource constraints that exist in developing countries, conducting a trial comparing the OPRA with the RAPIDO protocol in a setting of a wait‐and‐watch approach or similar trial that investigates SCRT as a modality of TNT is the need of the hour.

The major limitation of this study is its retrospective design which introduces a significant selection bias. While we have attempted to reduce this bias by means of propensity matching, certain confounding factors cannot be accounted for. This is exemplified by the statistically significant difference in rates of adoption of MIS and sphincter preservation surgery between the two groups despite propensity matching. Additionally, the shorter follow‐up in the SCRT cohort may underestimate late recurrences. While this study examined patients with high‐risk rectal cancer, in whom recurrence usually occurs within 2 years, the actual impact that late recurrences would have on the DFS and OS will only be evident on further follow‐up.

## 5 Conclusion

In a high‐volume center routinely performing extended TME resections with acceptable rates of CRM positivity, equivalent short‐term and long‐term outcomes can be achieved with SCRT compared to LCRT in most low rectal cancers. However, a subset analysis of clinical T4 tumors revealed inferior DFS with SCRT, highlighting the need for caution when selecting SCRT in cT4 patients. These findings suggest that while SCRT remains a viable and resource‐efficient strategy in selected patients, particularly in resource‐constrained settings, LCRT may be preferable in clinical T4 tumors until further prospective validation becomes available.

## Conflicts of Interest

The authors declare no conflicts of interest.

## Synopsis

This propensity‐matched retrospective study from a high‐volume tertiary cancer center compared short‐course radiation therapy (SCRT) with long‐course chemoradiation (LCRT) in patients with low rectal cancer. The study found no significant difference in disease‐free survival, overall survival, or local recurrence rates between the two modalities, suggesting SCRT is a viable alternative in selected patients. However, in clinical T4 tumors, SCRT was associated with significantly worse disease‐free survival, indicating LCRT may be preferable for this subgroup.

## Data Availability

Data is available on request from the authors.
